# Socioeconomic Distress as a Predictor of Missed First Outpatient Newborn Visits

**DOI:** 10.7759/cureus.14132

**Published:** 2021-03-26

**Authors:** Jimmy Yao, Adam T Perzynski, Yasir Tarabichi, Namita Swarup, Aparna Roy

**Affiliations:** 1 Pediatrics, Case Western Reserve University School of Medicine, Cleveland, USA; 2 Epidemiology and Public Health, MetroHealth System, Case Western Reserve University School of Medicine, Cleveland, USA; 3 Internal Medicine and Research Informatics, MetroHealth Medical Center, Cleveland, USA; 4 Pediatrics, MetroHealth Medical Center, Cleveland, USA

**Keywords:** no-shows, area deprivation index (adi), newborn and child health, first year visits, health care disparities, well child visits, socio-economic factors

## Abstract

Objective

To determine if the Area Deprivation Index (ADI) can serve as a predictor for patients from geographic regions of high socioeconomic distress as high risk for having no-shows to first-year newborn visits.

Methods

We assessed the no-show rate per patient from a large public safety-net hospital in Cleveland, Ohio, and the ADI of the census-designated tract for each patient's home geographic identifier (GEOID), aggregated into quintiles, and calculated differences in no-show-rates across census-designated tracts of different ADIs.

Results

A total of 2944 newborns from an approximate 18-month follow-up period between 2015-2017 were included. Large differences in no-shows per individual patient record (chi-square = 225, p = <0.001, df = 4) were found across quintiles of ADI. Heat-mapping indicated that census tracts with the highest ADIs and highest rates of no-show appointments encompass Cleveland's inner-city region.

Conclusion

The ADI is demonstrated to identify communities at high risk of no-show newborn appointments. Mapping these communities and their socioeconomic distress levels represented by ADI and missed appointment rate for each community can provide future direction for interventions targeted towards these communities to reduce no-show rates and improve overall community infant health.

## Introduction

The American Academy of Pediatrics (AAP) recommends following up pediatric newborns within 3-5 days after discharge from the hospital [1]. Outpatient newborn visits are essential for monitoring each newborn's weight, feeding, safety, and social determinants of a family's health [2]. Lack of continuity of follow up in a newborn can be related to noncompliance with medical appointments further in childhood [3]. Missed visits and loss-to-follow-up have significant consequences for the health of the newborn and the overall health of the community. By missing well-child visits early in life, children do not receive critical preventative care (e.g., vaccinations and screenings), and families miss an opportunity for health education with pediatric care providers [4].

Barriers to newborn outpatient visits have been described in the literature. Care delivery in urban low-income households faces significant challenges, which start as early as facing difficulty in completing guideline-based preventive care as newborns. A prior study found that infants in families with food insecurity and housing instability are at a higher risk for "no show visits" and overall missed appointments [5]. Both demographic and social data regarding the risk of missed appointments have been extensively studied, with various factors shown to be associated with a higher risk, such as being on Medicaid and lacking a car in the household [6,7]. Primary care is known to reduce health disparities; thus, challenges as early as completing primary care for newborns no doubt contribute to an ongoing risk of morbidity and mortality in children and impacts widening of health inequities [8]. However, a more complete understanding of social factors necessitates an examination of the influence of neighborhood-level social factors not regularly assessed in routine clinical settings. With the majority of physicians not feeling confident to address social determinants of health in clinical practice [9], an examination of neighborhood social indicators based upon patient addresses offers an opportunity to extend the value of readily available population health data.

The Area Deprivation Index (ADI) has been highlighted as a useful tool for measuring socioeconomic distress in a composite manner. The ADI is a compilation of multiple socioeconomic factors, such as the number of households without a vehicle, poverty level, level of education for individuals in each community, and single-parent households, aggregated to a census tract level [10]. The ADI has also been implemented in electronic health records (EHRs) as an efficient way to assess neighborhood-level determinants of a patient's health outcomes otherwise not available on clinical charts, providing a standardized measurement of social determinants while decreasing the data collection burden for frontline healthcare workers [11]. Prior studies have examined the association of the ADI and similar neighborhood measures with hospital readmissions, cardiovascular outcomes, lung cancer and other health conditions [12-15]. To our knowledge, there has been no study to date examining the relationship between ADI and no-show appointment rates in newborns. This study aims to assess the strength of the ADI, an area-based measure of socioeconomic distress, as a predictor of "no-show" appointments.

## Materials and methods

We conducted a retrospective observational study utilizing EHR data from an urban safety-net care system in Northeast Ohio. This project was approved by the MetroHealth Institutional Review Board. All analyses were conducted using R, version 3.5.0 (R Foundation for Statistical Computing, 2018).

As the public safety-net system of Cuyahoga County, MetroHealth provides service to a racially and ethnically diverse population, including a large proportion of African Americans, and rapidly growing Latino and refugee populations. Every year approximately 3000 new mothers are discharged from the maternity ward at MetroHealth with instructions to schedule a follow-up exam within one week. Mothers without an identified primary care provider (PCP) are scheduled to return to a MetroHealth facility for their child's first pediatric appointment. About 2400 visits are scheduled annually. Patients who elect to follow-up with their PCP outside the MetroHealth system do not get a scheduled follow-up exam. However, as many as half of these patients do not return for their scheduled appointment and are lost to follow up. Based on anecdotal evidence and records from nearby hospitals, it is unlikely that these patients are receiving any primary care during their first year of life.

Appointment status data was extracted from the EHR of newborn patients for each newborn's first-year well-child visits from an approximate 18-month follow-up period between 2015-2016 (n = 2944). Maternal addresses were geocoded to the census tract level via utilization of the geographic identifier (GEOID) of each address. Data from the American Community Survey (2016) was compiled into the ADI described by Singh [10] and implemented in the open source Sociome R package (https://cran.r-project.org/web/packages/sociome/index.html) [16]. The Sociome package implements a localized ADI estimation procedure by extracting principal components for the areas selected (in our case, we used Ohio census tracts as the reference region). Those component weights are utilized to then create an ADI score for each area across fifteen ADI indicators (we note that two items from Singh’s 1990 ADI, about the absence of indoor plumbing and the absence of a telephone are not included in our index due to these items having zero variation; all units have plumbing and telephone in Northeast Ohio). Other published research in our region has demonstrated that EHR-derived age cohorts had similar representations to Census estimates, even within ADI-quintiles [17]. Maternal records were divided into quintiles based on ADI.

Kruskal-Wallis tests were conducted to examine the differences in the number of no-show well-child appointments per individual patient record according to ADI quintile. The relationship of each quintile was graphed into a Violin Plot.

The rate of missed appointments per infant patient and ADI was aggregated at the census tract level and visualized on a regional heat-map.

## Results

**Figure 1:**
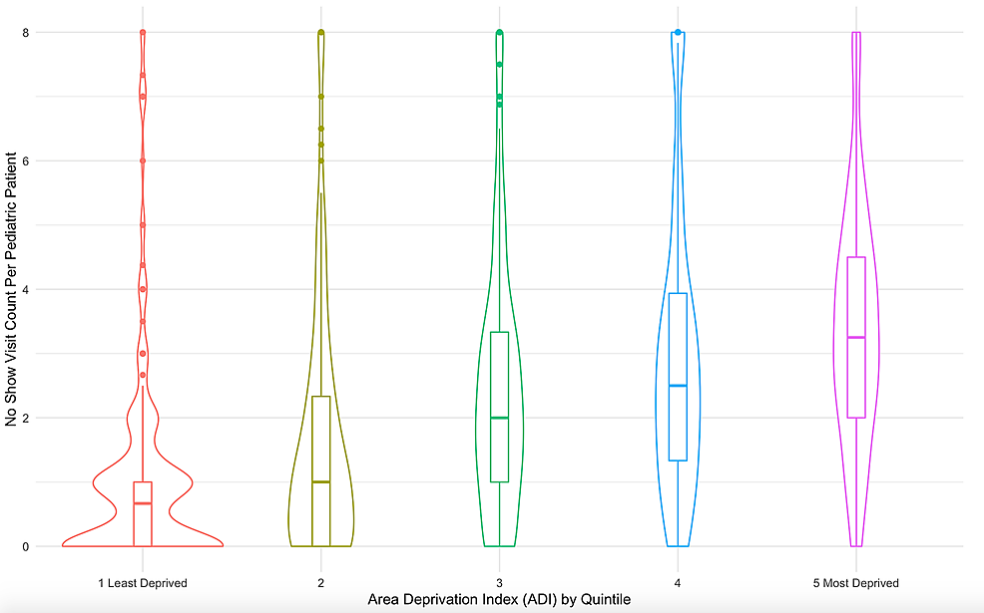
Violin Plot for No Show Visit Count Per Pediatric Patient v.s. Area Deprivation Index Quintile for MetroHealth Patients born between 2015-2016 in the Greater Cleveland Area ADI: Area Deprivation Index

No show visits per patient were significantly higher in patients residing in areas of highest socioeconomic distress (chi-square = 225, p = <0.001, df = 4), shown as quintiles of ADI (Figure 1). The mean ADI and mean number of no-show well-child appointments per individual patient record for each ADI quintile are shown in Table 1.

**Table 1:** ADI and No-Show Rate per Quintile ADI: Area Deprivation Index; SD: Standard Deviation

	Overall	Quintile 1	Quintile 2	Quintile 3	Quintile 4	Quintile 5
Mean ADI (SD)	106.36 (22.05)	76.57 (8.62)	92.67 (3.37)	105.04 (3.92)	119.8 (4.65)	137.8 (8.18)
Mean number of no-show well-child appointments per child (SD)	2.42 (2.60)	1.07 (1.64)	1.78 (2.31)	2.50 (2.08)	3.16 (3.03)	3.56 (2.85)

**Figure 2:**
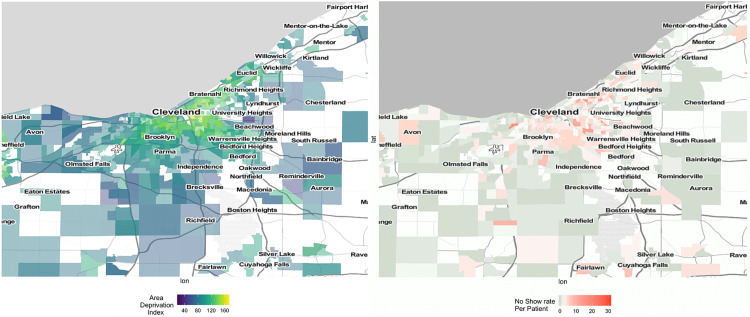
2016 US Area Deprivation Index and 2015-2016 No Show Rate per Child by Tract in the Greater Cleveland Area

Heat-mapping indicated that census tracts with the highest ADI scores and highest rates of no-show appointments encompass the inner-city region of Cleveland, Ohio and a few inner-ring suburbs geographically (Figure 2). These heat map trends also show neighborhood-level similarities to maps of the infant mortality rate for our region [18,19].

## Discussion

Our study shows that neighborhood socioeconomic position (measured by ADI) is a strong predictor of having no-shows to newborn appointments, especially for communities at high levels of socioeconomic distress. Higher no-show rates are associated with worse health outcomes in newborn and pediatric populations. Our findings further suggest that the processes through which neighborhood socioeconomic circumstances influence no-show rates for newborns should be further explored to understand additional downstream effects (e.g. infant mortality and serious illness).

A limitation of this study was that it explored the use of ADI and no-show appointment rates for a limited geographic region. Future studies are needed that combine multi-center populations that are more representative of the general population. Other limitations included the inability to assess interpersonal factors that could impact a newborn's likelihood of returning for routine visits. For example, having an inflexible schedule, difficulty with rescheduling appointments, perceived distrust of vaccinations, and a lack of communication with healthcare providers are all factors that are associated with missed appointments but are not addressed by the ADI [11]. In addition, a closer examination of how specific social needs vary for patients across urban neighborhoods could enable more targeted screening and social care referral efforts.

Higher levels of socioeconomic distress are correlated with higher levels of infant mortality, and this finding becomes especially apparent in a study that compared areas in a city of the highest socioeconomic status with those with the lowest socioeconomic status [20]. Another study indicated that specific socioeconomic factors, such as maternal marital status, education, and age, predicted infant mortality rate [21]. The ADI has the benefit of encompassing all these factors in some form in a composite manner. Population health management approaches seeking to improve appointment status, improve the health of newborns, and reduce socioeconomic disparities could likely benefit from using the ADI as an informative predictor and tool for targeting additional resources to high need communities and families, thereby preventing adverse newborn outcomes, while also maximizing limited resources and time available to healthcare providers.

## Conclusions

Mapping these communities and their socioeconomic distress levels represented by their ADI and the missed appointment rate for each community can provide future direction for interventions targeted towards these communities to reduce no-show appointment rates and thus improve overall community infant health one newborn visit a time.
